# No association of the dopamine D2 receptor genetic bilocus score (rs1800497/rs1799732) on food addiction and food reinforcement in Chilean adults

**DOI:** 10.3389/fnbeh.2023.1067384

**Published:** 2023-03-31

**Authors:** Nicole Hidalgo Vira, Karina Oyarce, Macarena Valladares Vega, Gary S. Goldfield, Enrique Guzmán-Gutiérrez, Ana M. Obregón

**Affiliations:** ^1^Escuela de Tecnología Médica, Facultad de Medicina y Ciencia, Universidad San Sebastián, Concepción, Chile; ^2^Escuela de Nutrición y Dietética, Facultad de Ciencias para el Cuidado de la Salud, Universidad San Sebastián, Concepción, Chile; ^3^Escuela de Terapia Ocupacional, Facultad de Salud y Ciencias Sociales, Universidad de las Ámericas, Santiago, Chile; ^4^Children’s Hospital of Eastern Ontario Research Institute, Ottawa, ON, Canada; ^5^Pregnancy Diseases Laboratory, Department of Clinical Biochemistry and Immunology, Faculty of Pharmacy, Universidad de Concepción, Concepción, Chile

**Keywords:** food addiction, polymorphisms, eating behavior, dopamine, food

## Abstract

**Purpose:** Different systems regulate food intake. In the reward system, dopamine (DA) is the main neurotransmitter, and a variety of genetic variants (rs1799732 and rs1800497) are associated with addiction. Addiction is a highly polygenic disease, where each allelic variant adds a small amount of vulnerability. Polymorphisms rs1799732 and rs1800497 are associated with eating behavior and hedonic hunger, but links to food addiction remain unclear.

**Aim:** To evaluate the association between the bilocus profile (rs1799732-rs1800497) of the dopaminergic pathway with food reinforcement and food addiction in Chilean adults.

**Methods:** A cross-sectional study recruited a convenience sample of 97 obese, 25 overweight, and 99 normal-weight adults (18–35 years). Anthropometric measurements were performed by standard procedures and eating behavior was assessed using the: Food Reinforcement Value Questionnaire (FRVQ) and Yale Food Addiction scale (YFAS). The DRD2 genotypes were determined by TaqMan assays (rs1800497 and rs1799732). A bilocus composite score was calculated.

**Results:** In the normal weight group, individuals who were heterozygous for the rs1977932 variant (G/del) showed higher body weight (*p*-value 0.01) and abdominal circumference (*p*-value 0.01) compared to those who were homozygous (G/G). When analyzing rs1800497, a significant difference in BMI was observed for the normal weight group (*p*-value 0.02) where heterozygous showed higher BMI. In the obese group, homozygous A1/A1 showed higher BMI in comparison to A1/A2 and A2/A2 (*p*-value 0.03). Also, a significant difference in food reinforcement was observed in the rs1800497, where homozygous for the variant (A1A1) show less reinforcement (*p*-value 0.01).In relation to the bilocus score in the total sample, 11% showed “very low dopaminergic signaling”, 24.4% were “under”, 49.7% showed “intermediate signaling”, 12.7% showed “high” and 1.4% showed “very high”. No significant genotypic differences were observed in food reinforcement and food addiction by bilocus score.

**Conclusions:** The results indicate that the genetic variants rs1799732 and rs1800497 (Taq1A) were associated with anthropometric measurements but not with food addiction or food reinforcement in Chilean university students. These results suggest that other genotypes, such as rs4680 and rs6277, which affect DA signaling capacity through a multilocus composite score, should be studied. Level V: Evidence obtained from a cross-sectional descriptive study.

## Introduction

Obesity is a chronic disease characterized by increased body fat. Obesity is an important risk factor for chronic non-communicable diseases (CNCD), pathologies that have been strongly positively associated with body mass index (BMI: kg/m^2^; Skinner et al., 2018). In Chile, 60% of the population over 15 years of age has some degree of excess weight (Ministerio de salud, 2018). For a normal body weight balance, there must be an equilibrium in the body energy equation. It is well documented that the two main factors that influence the development of obesity are a decrease in energy expenditure and/or overconsumption of food. This last factor is primarily related to individuals’ eating behavior which has shown an important genetic contribution (Carnell et al., 2008). In this sense, twin approaches are based on the estimation of genetic and environmental influence on the variation of any measured trait (for example, eating behavior) and have shown a strong heritability for the traits “satiety responsiveness” and “food responsiveness” (63%–75%; Carnell et al., 2008).

In relation to eating behavior, food addiction is characterized by obsessive-compulsive consumption of certain foods and persistent attempts to reduce or control food intake, especially those highly processed foods (HP) refer to industrially created foods that contain unnaturally high levels of refined carbohydrates (e.g., sugar and white flour) and/or fat (Schulte et al., 2015; Gearhardt et al., 2016; Parnarouskis et al., 2022). Moreover, there is also the concept of reinforcing value, defined as how much work is required to gain access to foods that are considered to be reinforcing (stimulus) is especially of interest. Both behaviors have been associated with increased energy intake (Temple et al., 2009). To date, there have been some polymorphisms and mutations within genes primarily associated with appetite, metabolism, and eating behavior (Lindgren et al., 2018), and so far, 127 loci have been found within the human genome linked to BMI (Singh et al., 2017). The mechanisms underlying these behavioral disorders are largely unknown, and some authors have studied different genetic variations and the role of miRNAs in the development of food addiction (García-Blanco et al., 2022).

Food intake is regulated by two mechanisms: the homeostatic system, which regulates energy balance, and the non-homeostatic system, which primarily activates pleasure and reward circuits in the central nervous system (CNS) in response to highly palatable foods (Lindgren et al., 2018). In this system, foods with high palatability and high energy density stimulate the release of DA in the brain, and the level of DA released correlates with the pleasure experienced from eating (Singh et al., 2017). A way to indirectly study individual differences in DA levels in the brain is to look at polymorphisms in genes that constitute the non-homeostatic system of intake (a system that makes eating highly palatable foods enjoyable), including DA receptors and transporters (Epstein et al., 2007).

There is evidence that certain variations of genes that are part of the brain reinforcement system, such as the dopamine receptor 2 (DRD2) and the dopamine receptor 4 (DRD4), have been linked to addiction-related behaviors, with a decrease in the number of DA receptors and/or an increase in the synaptic catabolism (Fattore et al., 2014).

In the dopaminergic pathway, Taq1A (rs1800497) is one of the most studied single nucleotide polymorphisms (SNPs). This variant is found in exon 8 of a gene adjacent to DRD2 (ANKK1: Ankyrin repeat and kinase domain containing 1), it changes glutamate to lysine at codon 713 (Céspedes, 2017) and has been linked to a lower receptor density (Blum et al., 2018), self-regulation disorders, excessive consumption of food and loss of control over dietary intake (Ariza et al., 2012) and obesity and addiction (McDonell et al., 2018). A similar finding was reported indicating that obese heterozygous for the variant (A1/A2) were more likely to experience difficulties with weight loss and maintenance (Winkler et al., 2012). Another study also reported a greater level of activation in brain areas sensitive to DA in carriers of the A1 allele, particularly in individuals with a higher body mass index (BMI: kg/m^2^; Stice et al., 2008).

In adults, our group showed that there was no relationship between food addiction and the Taq1A variant in DRD2. However, when stratified by sex and nutritional status, obese female carriers of the A1 allele reported greater scores on emotional eating and snack food reinforcement compared to non-carriers (Obregón et al., 2022).

The -141C Ins/del polymorphism (rs1799732) in the DRD2 gene, which results in the depletion of one of two cytosines, has been shown to be associated with addiction. It has been associated with several CNS disorders, including alcohol dependence, suicide attempts, and psychiatric disorders (Feistauer et al., 2018), and the associated mechanism is a decreased expression of DRD2 (Arinami et al., 1997).

The disadvantage of the assessment of an isolated polymorphism is that a single genetic variant contributes only a small fraction of the phenotypic variance of a trait, and therefore its independent effects are usually not significant (Davis et al., 2013). To address this, the literature has described a novel and more robust approach suggesting multilocus genetic profiling (MLGP) scoring. Using this approach, it has been found that future weight gain is positively correlated with higher MLGP in adults (Taq1A2/A2, COMT Val/Val, DRD2 -141C Ins/De, DRD4-S, DAT1-S; Yokum et al., 2015). Also, in adolescents, it was shown a positive correlation between MLGP and less responsiveness of reward regions in response to anticipated receipt of tasty food (Stice et al., 2012). Finally, an association was found between MLGP and food addiction, binge eating, and food cravings in adults (Davis et al., 2013).

We have two main objectives in the present study. Our first objective was to determine whether anthropometric measurements differed based on genotype. We hypothesized that carriers of variants rs1799732 and rs1800497 would have higher BMIs and anthropometric measurements. A second objective was to examine the association between the bilocus score and food reinforcement and food addiction prevalence. It was hypothesized that individuals with higher bilocus scores would exhibit higher food reinforcement and a higher prevalence of food addiction.

## Materials and methods

### Design and subjects

Our study, which used a cross-sectional design, was conducted between January 2016 and March 2017. The convenience sample consisted of 221 adults (74% females) aged 18–54, of whom 43.8% were obese, 11.3% were overweight, and 44.8% were normal weight. The participants were excluded if they were participating in a weight loss program, were underweight, on medications that affected body weight or metabolism, had diabetes, weight-related hepatic or renal disease, or did not have complete phenotypic data. We recruited participants from diverse sources in the community, such as university campuses, community centers, and from the Universidad San Sebastian website^1^. All participants signed informed consent forms and were tested in a laboratory at San Sebastian University. This study was approved by the Research Scientific Ethics Committee of San Sebastián University at the institution of the lead author. In order to conduct the study, the ethics guidelines set forth in the Declaration of Helsinki were followed.

### Anthropometry

Adults were evaluated for weight and height using a SECA scale with a height rod included, with a sensitivity of 100 g and 0.5 cm. Furthermore, bioimpedance measurements were performed using the TANITA 300. The height was measured using the Frankfort plane. BMI was calculated as the ratio of weight (in kilograms) divided by the square of height in meters (kg/m^2^; Gordon et al., 1991). The classification of nutritional status was carried out using the criteria proposed by the WHO (Pi-Sunyer et al., 1998).

### Eating behavior

We used two questionnaires: (a) **Food Addiction Scale (YALE-FAS)**, which has been validated for use by Obregón et al. (2015) for the Chilean adult population. Based on the DSM-IV criteria for substance dependence, this survey consists of 25 questions regarding eating behavior for the past 12 months, as well as mixed response categories. In addition, it contains two questions that are intended to assess the presence of clinical alteration or distress. The diagnostic criterion is fulfilled when three symptoms are present and clinical alteration or distress is observed (Gearhardt et al., 2009). (b)** Food Reinforcement Value Questionnaire (FRVQ)**: The Food Reinforcement Value Questionnaire (FRVQ) was validated by Hill et al. (2009, 2020). In the assessment, 12 items are asked related to the efforts that individuals are willing to make to get a specific reinforcer (most preferred palatable snack food or their highest rated healthy alternative fruits/vegetables). The reinforcing value of the food is defined as the amount of work a participant is willing to do to obtain food relative to alternative reinforcers, with higher scores reflecting greater food reinforcement for palatable foods.

The schedule of reinforcement began at an equal fixed ratio (FR) of 20 presses in the button on a joystick to receive either the snack food or no alternative fruit/vegetables [e.g., “Would you prefer to press the button 20 times for a biscuit (cookie) or 20 times for a preferred fruit?”] (Goldfield et al., 2005). For each following question, the number of button presses required for the snack food increased on a fixed ratio progressive schedule of reinforcement of 20 presses per question, resulting in a maximum VR of 20 or 240 presses required for fruit/vegetables or snack food at the last question. The RV of food was defined as the total number of snack food choices made. The proportion of snack food choices made for the schedule of reinforcement was calculated.

### Biological sampling

Blood samples (4 ml) were collected after an overnight fast (8 h) by venipuncture using a vacuum system in accordance with a standard protocol using EDTA-K3 as an anticoagulant. Plasma was separated from buffy coat and red blood cells after centrifugation at 3,300 rpm for 10 min.

### DNA extraction

Genomic DNA was extracted using the QIAAMP DNA blood mini kit (#51106 250 determinations) from QIAGEN and stored at −20°C according to manufacturer instructions.

### Molecular genotyping

#### Genetic variant rs1800497

This variant was evaluated by PCR-RFLP assay in previous work (Obregón et al., 2022). The presence or absence of a given allele was established based on the predicted sizes of the PCR products: for homozygous A1/A1, one band of predicted size 307 bp was observed, for A1/A2 heterozygotes, three bands of predicted size 307 bp, 177 bp, and 127 bp were observed, and for A2/A2 homozygotes, two bands of predicted size 177 base pairs (bp) and 127 bp were observed.

#### Genetic variant rs1799732

This variant was determined with a predesigned Taqman assay ID C__33641686_10 (Applied Biosystems), using a QuantStudio^TM^ 3 Real-Time PCR Systems following the manufacturer’s instructions. Homozygous G/G, heterozygous G/Del, and homozygous Del/Del genotype groups were defined.

#### Bilocus genetic profile

We calculated individual Bilocus genetic profile scores using the two polymorphisms assessed of the dopaminergic system, which have previously been associated with alterations in the dopaminergic pathway. Across the two loci, Taq1A A2/A2 and Del/Del genotypes were assigned a score of 1, as they are considered to have a high dopaminergic signaling. Taq1A A1/A2 and G/Del genotypes were assigned a score of 0.5 and classified as having an “intermediate dopaminergic signaling”. Finally, A1/A1 and GG genotypes were assigned a score of 0, indicating a “low dopaminergic signaling”. The overall score for each participant at each locus will be 0–1, and for the total route a score of 0–2 (Nikolova et al., 2011).

#### Sample size calculation

The sample size was calculated using the following formula described by Mathew et al. 2003 for determining the prevalence of a certain disease with a total of 221 adults (Casal and Mateu, 2003).


$$\text{n=}\frac{{\text{z}}^{\text{2}}\text{pq}}{{\text{B}}^{\text{2}}}$$

Where, *n* = Sample size; *z* = 1.96 for 95% confidence; 2.56 for 99%; *p* = Expected frequency of the factor to be studied; *q* = 1-p; B = Accuracy or admitted error.

### Data analysis

A descriptive analysis was conducted to characterize the sample as mean or median and standard deviation. We estimated genotype and allele frequencies and evaluated the Hardy-Weinberg equilibrium using the goodness-of-fit X^2^ test. An examination of the differences and associations between groups was conducted using non-parametric statistics (Mann-Whitney and Kruskal-Wallis tests), including a sex-specific analysis. Data were analyzed in STATA 14.0 software.

## Results

### Association between the genetic variants and anthropometric measure

The sample was composed of 221 adults (74% female and 25% male): 45% normal weight range, 11% were overweight, and 44% were with obesity. By nutritional status, we found a significant difference in weight, BMI, % body fat, waist-to-height index, and abdominal circumference. The descriptive results of food reinforcement and food addiction by gender and nutritional status were previously reported (Obregón et al., 2022).

The genotypic frequency of rs1799732 was 73.1% for the GG genotype, 25.5% for G/Del, and 1.4% for Del/Del. For the rs1800497, the genotypic frequency was 56.4% for A2A2, 33.6% for A1A2, and 9.9% for A1A1. Both variants meet the Hardy-Weinberg equilibrium (*p*-value = 0.7 and 0.05, respectively).

Table 1 shows anthropometric measurements by genotype of rs1799732 and rs1800497 variants.

**Table 1:** Anthropometric measurements by genotype of rs1799732 and rs1800497 variants.

	Normal weight				Overweight				Obesity			
	rs1799732				rs1799732				rs1799732			
	G/G	G/Del	Del/Del		G/G	G/Del	Del/Del		G/G	G/Del	Del/Del	
	(*n* = 73)	(*n* = 23)	(*n* = 1)	*p*-value	(*n* = 21)	(*n* = 4)		*p*-value	(*n* = 61)	(*n* = 27)	(*n* = 2)	*p*-value
	Mean ± SD	Mean ± SD	Mean ± SD		Mean ± SD	Mean ± SD	Mean ± SD		Mean ± SD	Mean ± SD	Mean ± SD	
	rs1799732				rs1799732				rs1799732			
Weight (kg)	59.3 ± 6.3*	63.6 ± 7*	51.5 ± 0	0.01*	72.5 ± 10.8	70.7 ± 6.2	-	0.6	90.6 ± 12.0	91.4 ± 11.5	97.9 ± 28.2	0.8
Height (m)	1.63 ± 0.07	1.66 ± 0.08	1.58 ± 0	0.1	1.65 ± 0.11	1.58 ± 0.03	-	0.1	1.65 ± 0.08	1.62 ± 0.08	1.66 ± 01.1	0.6
BMI (kg/mt^2^)	22.3 ± 1.52	23.0 ± 1.53	20.6 ± 0	0.06	26.5 ± 2.3	28.3 ± 2.1	-	0.1	33.4 ± 3.9	34.8 ± 3.6	35.2 ± 6.23	0.2
Weight to height ratio	0.46 ± 0.04	0.48 ± 0.03	0.42 ± 0	0.1	0.53 ± 0.0	0.55 ± 0.06	-	0.5	0.64 ± 0.07	0.64 ± 0.09	0.68 ± 0.02	0.5
Abdominal circumference (cm)	75.5 ± 3.34*	79.24 ± 6.32*	67 ± 0	0.01*	87.6 ± 8.5	87.5 ± 9.9	-	0.9	105.2 ± 10.6	102.5 ± 12.8	112.5 ± 9.1	0.2
Body fat %	24.3 ± 6.04	23.9 ± 6.88	20.30 ± 0	0.7	26.5 ± 9.4	35.8 ± 3.3	-	0.05	40.18 ± 7.7	42.3 ± 6.11	43.2 ± 2.7	0.4
	rs1800497				rs1800497				rs1800497			
	A2/A2	A1/A2	A1/A1		A2/A2	A1/A2	A1/A1		A2/A2	A1/A2	A1/A1	
	(*n* = 54)	(*n* = 32)	(*n* = 11)	*p*-value	(*n* = 15)	(*n* = 8)	(*n* = 2)	*p*-value	(*n* = 49)	(*n* = 31)	(*n* = 8)	*p*-value
	Mean ± SD	Mean ± SD	Mean ± SD		Mean ± SD	Mean ± SD	Mean ± SD		Mean ± SD	Mean ± SD	Mean ± SD	
Weight (kg)	59.6 ± 7.11	61.3 ± 6.6	60.4 ± 5.6	0.3	73.3 ± 8.7	68.0 ± 12.4	80.4 ± 6.5	0.2	90.7 ± 12.1	90.0 ± 13.5	96.4 ± 3.8	0.1
Height (m)	1.64 ± 0.07	1.63 ± 0.08	1.62 ± 0.06	0.9	1.64 ± 0.1	1.62 ± 0.09	1.73 ± 0.02	0.3	1.64 ± 0.08	1.63 ± 0.09	1.62 ± 0.07	0.6
BMI(kg/mt^2^)	22.1 ± 1.5*	23.0 ± 1.4*	22.8 ± 1.3	0.02*	27.3 ± 1.6	25.9 ± 3.5	27.0 ± 1.5	0.6	33.6 ± 4*	33.6 ± 3.3†	36.8 ± 2.7*†	0.03*
Weight to height ratio	0.46 ± 0.04	0.48 ± 0.04	0.48 ± 0.04	0.07	0.54 ± 0.05	0.53 ± 0.07	0.51 ± 0.03	0.5	0.64 ± 0.08	0.63 ± 0.06	0.70 ± 0.08	0.1
Abdominal circumference	75.1 ± 6.4	77.8 ± 6.8	77.9 ± 4.8	0.1	88.9 ± 7.5	85.0 ± 11.1	87.7 ± 6.0	0.9	104.7 ± 11.9	102.0 ± 9.4	113.1 ± 11.7	0.07
Body fat %	23.2 ± 6.1	25.1 ± 6.1	26.1 ± 6.2	0.1	28.1 ± 9.7	29.6 ± 9.1	20.5 ± 5.7	0.5	40.6 ± 7.9	40.7 ± 6.2	44.5 ± 6.2	0.1

*Show significant differences between A1A1 and A2A2. ^†^Show significant differences between A1A1 and A1A2.

In the normal weight group, individuals who were heterozygous for the rs1977932 variant showed higher body weight (*p*-value 0.01) and abdominal circumference (*p*-value 0.01). In the overweight group, heterozygous G/del showed a higher percentage of body fat, however, it did not reach statistical significance (26.5 ± 9.4; 35.8 ± 3.3 *p*-values of 0.05). In the obese group, there were no differences in the anthropometric measures. When analyzing rs1800497, a significant difference in BMI was observed for the normal weight group (*p*-value 0.02) where heterozygous showed higher BMI. In the obese group, homozygous A1/A1 showed higher BMI in comparison to A1/A2 and A2/A2 (*p*-value 0.03).

In the normal group of female carriers of the A1 allele, we found an increase in BMI, weight-to-height ratio, abdominal circumference, and total body fat mass. It was found that those who carried the A1 allele had a higher percentage of food choices in the obesity group. Obese males with the del-allele had a significant increase in % body fat mass. In addition, male carriers of the A1 allele in the obesity group had a higher body mass index (Supplementary Tables 1 and 2).

Table 2 shows food reinforcement by genotype and nutritional status. No significant difference in food reinforcement was observed when analyzing rs1799732 in the total sample, nor when stratifying by nutritional status and gender.

**Table 2:** Food reinforcement by the genotype of rs1799732, rs1800497, nutritional status, and gender in Chilean university students.

	rs1799732				rs1800497			
	G/G	G/Del	Del/Del		A2/A2	A1/A2	A1/A1	
	(*n* = 155)	(*n* = 54)	(*n* = 3)	*p*-value	(*n* = 118)	(*n* = 73)	(*n* = 21)	*p*-value
	Mean ± SD	Mean ± SD	Mean ± SD		Mean ± SD	Mean ± SD	Mean ± SD	
Food reinforcement (*n* = 212 all sample)	19.4 ± 23.0	17.2 ± 20.7	11.1 ± 4.8	0.7	17.37 ± 21.4†	22.8 ± 23.1*†	13.8 ± 23.6*	0.01†
Normal weight	17.3 ± 20.2	14.8 ± 18.8	8.3 ± 0	0.6	14.9 ± 17.2	19.5 ± 20.4	16.6 ± 28.8	0.2
Overweight	22.2 ± 27.3	8.3 ± 11.7	-	0.3	25.0 ± 30.3	15.6 ± 15.7	0 ± 0	0.2
Obesity	21.0 ± 24.8	20.6 ± 23.0	12.5 ± 5.8	0.9	17.6 ± 22.3	28.2 ± 26.5	13.5 ± 18.3	0.05
Women	21.2 ± 23.9	18.8 ± 22.3	8.3 ± 0	0.5	17.8 ± 21.7	25.1 ± 24.8	18.2 ± 25.6	0.05
Men	14.3 ± 19.7	13.3 ± 15.6	16.6 ± 0	0.8	16.1 ± 2.9†	15.1 ± 14.0*	0 ± 0*†	0.03*

*†Significant differences were analyzed with the nonparametric Kruskal-Wallis and dun test by genotype. Groups with the same symbol reflects significant differences between them.

When analyzing rs1800497, a significant difference in food reinforcement is observed, where individuals homozygous for the variant (A1A1) show less reinforcement (*p*-value 0.01). This difference is given mainly by the male gender (*p*-value 0.03).

### Association between genotype, food categories, and food addiction

Among food categories, chocolate, candies, bread, rice, chips, pretzels, French fries, bacon, hamburgers, and soda consumption were significantly associated with food addiction (*p* < 0.05 test η^2^; Supplementary Table 3).

Table 3 shows the prevalence of food addiction by genotype (rs1799732/rs1800497), nutritional status, and gender. No significant difference was observed according to genotype. When we stratified the sample by nutritional status we observed that in the overweight group, the heterozygotes for the rs1799732 variant have a higher prevalence of addiction (*p*-value 0.001). Additionally, men show a higher prevalence of addiction (*p*-value: 0.01). For rs1800497, no significant difference was observed in the food addiction prevalence.

**Table 3:** Food addiction by the genotype of rs1799732 and rs1800497, nutritional status and gender in Chilean university students.

rs1799732	G/G	G/Del	Del/Del
	(*n* = 155)	(*n* = 54)	(*n* = 3)	*p*-value
Food Addiction diagnosis	31/155 (20%)	13/54 (24%)	1/3 (33.3%)	0.71
Normal weight	5/73 (6.8%)	1/23(4.3%)	0/1 (0%)	0.88
Overweight	1 /21(4.7%)	3/4 (75%)	-	0.001*
Obesity	25/61 (40.9%)	9/27 (33.3%)	1/2(50%)	0.75
Women	27/115 (23.4%)	12/39 (30.7%)	0/2 (0%)	0.47
Men	4/40(10%)	1/15 (6.67%)	1/1 (100%)	0.01*
rs1800497	A2/A2	A1/A2	A1/A1	
	(*n* = 118)	(*n* = 71)	(*n* = 21)	*p*-value
Food Addiction diagnosis	21/119 (17.6)	18/71 (25.3)	6/21 (28.5)	0.31
Normal weight	2/54 (3.7)	2/32 (6.25)	2/11 (18.1)	0.19
Overweight	3/15 (20.0)	1 /8 (12.5)	0 /2 (0.0)	0.72
Obesity	16/50 (32)	15/31 (48.3)	4/8 (50)	0.26
Women	18/85 (21.1)	15 /55(27.2)	6/16 (37.5)	0.34
Men	3/34 (8.82)	3/16 (18.0)	0/5 (0.0)	0.4

*Statistically significant (*p* < 0.05; chi-square test).

### Association between the bilocus score and food addiction

Table 4 shows the bilocus frequency in the total sample. In the total sample, 11.7% showed “very low dopaminergic signaling”, 24.4% were “under”, 49.7% showed “intermediate signaling”, 12.7% showed “high”, and 1.4% showed “very high”. Food reinforcement is shown in Table 5 by the bilocus score. There were no significant differences when we observed food reinforcement by the different bilocus groups. Food reinforcement was stratified by gender and nutritional status and no differences were observed.

**Table 4:** Frequency of Score Bilocus (rs1799732 and rs1800497) in Chilean university students.

Frequency Score Bilocus (rs1799732 and rs1800497)					
	0	0.5	1	1.5	2
	“very low”	“under”	“intermediate”	“high”	“very high”
	*n* = 26	*n* = 54	*n* = 110	*n* = 28	*n* = 3
Total	11.7%	24.4%	49.7%	12.7%	1.4%

*Statistically significant (*p* > 0.05). Individual genetic profile scores represent the sum of ‘high’ DA genotypes across two functional polymorphic loci. ‘High’ genotypes received a score of 1, ‘low’ genotypes a score of 0, and ‘intermediate’ genotypes a score of 0.5. For example, the genetic profile score for an individual with the following two polymorphisms, DRD2 -141C Ins/Ins and DRD2 Taq1A (0 + 0).

**Table 5:** Food reinforcement by Score Bilocus (rs1799732 and rs1800497), nutritional status and gender in Chilean university students.

Frequency Score Bilocus (rs1799732 and rs1800497)						
	0	0.5	1	1.5	2
	“very low”*n* = 26	“under”*n* = 54	“intermediate”*n* = 110	“high”*n* = 28	“very high”*n* = 3	*p*-value
	Mean ± SD	Mean ± SD	Mean ± SD	Mean ± SD	Mean ± SD
Food reinforcement	19.2% ± 31.3%	24.2% ± 25.0%	17.4% ± 20.5%	17.9 ± 23.3	11.1 ± 4.8	0.16
*Gender*						
Women	23.8% ± 33.3%	27.1% ± 26.5%	17.4% ± 20.2%	20.0% ± 25.7%	8.3% ± 0%	0.17
Men	0% ± 0%	12.9% ± 13.1%	17.2% ± 21.5%	12.5% ± 16.1%	16.7% ± 0%	0.19
*Nutritional status*						
Normalweight	25.8% ± 37.6%	19.3% ± 20.5%	15.7% ± 17.5%	14.1% ± 19.0%	8.33 ± 0%	0.75
Overweight	0% ± 0%	19.4% ± 16.4%	23.9% ± 30.9%	12.5 ± 17.7%	-	0.36
Obesity	16.8% ± 27.4%	30.8% ± 30.0%	17.0% ± 19.9%	22.4% ± 28.1%	12.5 ± 5.9	0.30

*Statistically significant (*p* < 0.05; Kruskal-Wallis).

The prevalence of food addiction in the total sample is shown in Table 6. In the total sample and stratified by gender, no difference in prevalence was observed. Based on stratification by nutritional condition, we found that in the overweight high score group 2/2 participants were diagnosed with food addiction, but this did not reach statistical differences.

**Table 6:** Prevalence of food addiction by Bilocus score (rs1799732 and rs1800497), gender and nutritional status in Chilean university students.

Frequency Score Bilocus (rs1799732 and rs1800497)						
	0	0.5	1	1.5	2
	“very low”	“under”	“intermediate”	“high”	“very high”	*p*-value
Food Addiction diagnosis	*n* = 8/26 (30.8%)	*n* = 16 /54 (29.6%)	*n* = 17/110 (15.5%)	*n* = 7/28 (25%)	*n* = 1/· (33.3%)	0.12
colspan="7">*Gender*
Women	*n* = 8/21 (38.6%)	*n* = 14/43 (32.6%)	*n* = 14/79 (17.7%)	*n* = 7/20 (35%)	*n* = 0/2 (0%)	0.12
Men	*n* = 0/5 (0%)	*n* = 2/11 (18.1%)	*n* = 3/31(9.6%)	*n* = 0/8 (0%)	*n* = 1/1 (100%)	0.13
*Nutritional Status*						
Normalweight	*n* = 2/11(18%)	*n* = 2/25 (8%)	*n* = 1/49 (2.0%)	*n* = 1/13 (7.6%)	*n* = 0/1 (0%)	0.16
Overweight	*n* = 0/2 (0%)	*n* = 0/6 (0%)	*n* = 2/15 (13.3%)	*n* = 2//2 (100%)	*n* = 0/0 (0%)	0.05
Obesity	*n* = 6/13 (46.1%)	*n* = 14/23 (60.8%)	*n* = 14/46 (30.4%)	*n* = 4/13 (30.7%)	*n* = 1/2 (50%)	0.12

*Statistically significant (*p* < 0.05; Fisher exact).

The anthropometric measurements by risk score delivered by the Bilocus Score, showed higher weight, BMI, waist-to-height ratio, abdominal circumference, and body fat in the group categorized as “very high risk”, however, did not reach statistical significance.

## Discussion

In the present study, we evaluated the prevalence of rs1799732 and rs1800497 variants located in the dopaminergic pathway in a population of adults from Chile. In our study, the genotypic frequency for the rs1799732 variant was 25.5% for the heterozygous G/Del and 1.4% for the homozygous Del/Del. These results agree with other populations that have reported 24% of the total population heterozygous “G/Del” (study of five super populations: Africa, America, East Asia, Europe, and South Asia; Ensembl., 2021a, b). For the rs1800497 variant, previous reports have set in 56.4% for the A2/A2 homozygote, 33.6% for the A1/A2 heterozygote, and 9.9% for A1/A1 homozygote (Obregón et al., 2022). The objective of this study was to evaluate the association between the bilocus genetic score (rs1799732/rs1800497) belonging to the dopaminergic pathway, with food reinforcement and the prevalence of food addiction in adults.

The del-allele of the rs1799732 variant is significantly associated with lower DRD2 gene expression and promoter activity (Davis et al., 2013). In addition, this polymorphism has been associated with disorders associated with alcohol dependence (Prasad et al., 2010). The A1-allele of the rs1800497 has been associated with reduced DA binding sites in the brain, nicotine addiction, opioid dependence, and suicidal thoughts (Hill et al., 2009; Chen et al., 2011; Voisey et al., 2012). Moreover, obese individuals demonstrate reduced DRD2 availability in the striatum, similar to drug addicts (Blum et al., 2013).

In the present study, there was no association between the rs1799732 variant and food reinforcement and food addiction. These results are consistent with a study of 270 children who were followed from 12 to 16 months of age until 7–8 years of age, in which no association was found between the DRD2 -141C Ins/Del polymorphism (rs1799732) and food intake and anthropometric parameters.

In contrast, participants with the genotype A1/A1 had a higher BMI and A1/A2 carriers had greater reinforcement of food. A1 alleles were also associated with a higher intake of fat (Feistauer et al., 2018).

The Dopamine neurotransmitter has been strongly related to the rewarding process and has been associated with the motivation for food consumption. Specifically, it has been associated with food addiction, with increasing evidence suggesting that there is a neurological dysfunction in food addicts, similar to what occurs in people addicted to drugs. In this sense, the limbic and cortical regions involved in motivation, memory, and self-control are activated by gastric stimulation in subjects with obesity, similar to subjects addicted to drugs. This phenomenon can be explained due to a decrease in DA receptors in obesity and drug-addicted subjects. Moreover, it has been described that obese women who meet the criteria for food addiction, as compared to obese women who do not meet the criteria, exhibited a differential response to highly and minimally processed food cues, which is correlated with cravings in persons with substance abuse disorders (Schulte et al., 2019).

Performing rewarding activities, generate an immediate release of DA in the mesolimbic reward centers of the brain, and the hypodopaminergic functioning in obese patients may induce overeating in an effort to compensate for this reward deficit (Blum et al., 2013). In relation to this, it has been described that obese subjects have a lower response to food rewards compared to normal-weight subjects, probably because obese subjects have a lower density of DA receptors, which compromises DA signaling (Stice et al., 2008). These results are consistent with a study that reported greater hedonic hunger in participants carrying one risk allele for the rs1800497 variant (A1/A1 or A1/A2) and with at least one del-allele for the 1799732 variant (Del/Del or Del/ Ins) in overweight/obese participants (Aliasghari et al., 2021).

In our study, to assess addictive behavior to food, we used the Food Addiction Scale (YALE-FAS; Gearhardt et al., 2009). This tool has been previously validated in our population (Obregón et al., 2015) and has shown recent validity and good consistency with the DSM5 concept of substance use disorder (Gearhardt et al., 2016; Schiestl et al., 2022). Using the YALE-FAS it was observed that higher scores are associated with obesity, binge eating, impulsivity, and higher scores in the calculation of a Multi Locus Genetic Profile of the dopaminergic system (Davis et al., 2013). Previous data of our group estimated that 22.2% fulfil the criteria of food addiction (Obregón et al., 2022). In these senses, studies suggest that food addiction varies from 5% in the general population to more than 40% in obese people (Schiestl et al., 2022). A meta-analysis that evaluated food addiction using the Yale Food Addiction Scale found that 19.9% of the population met the criteria for food addiction, with the highest scores observed in adults older than 35 years, women, and overweight and obese participants (Pursey et al., 2014). Also, it has recently been described during the COVID-19 pandemic that the prevalence of food addiction was 19.1% in the Brazilian population and associated with depression and anxiety (da Silva Júnior et al., 2022).

Similarly, another study that included 652 participants observed that between 80% and 88% of individuals addicted to food were overweight or obese, supporting that the “addition to food” has contributed to the increase in the prevalence of obesity in the general population (Pedram et al., 2013).

Food reinforcement is defined as how much effort (value) a person is able to exert to gain access to food (stimulus). In our study, food reinforcement was evaluated through the FRVQ. This instrument measures how hard an individual is willing to work to obtain food that they consider reinforcing. In this sense, people for whom food has a high reward value tend to work harder to gain access to food compared to those with a low food reward value. In this relationship, it has been speculated that food may produce addictive behaviors similar to those associated with drug addiction. An example of this is sugar, which meets many of the general characteristics of addictions, such as binge eating behavior, escalation in the dose of sugar intake, and anxiety, among others (Epstein et al., 2011). In this sense, recently it has been reported that also sweet high-fat food and non-alcoholic sugary drink are related to emotions after consumption (Cummings et al., 2022).

We found no significant difference between FRV and nutritional status, however, higher FRV scores were observed in overweight individuals compared to normal-weight individuals, but did not reach a statistical difference. These results are consistent with previous studies where the eating behavior of 233 adults was evaluated and no difference was found with nutritional status, but with energy consumption (French et al., 2014).

Our study found no association between genetic polymorphisms, food reinforcement, and the prevalence of food addiction. This contrasts with what was observed by Epstein et al. (2007) who evaluated food reinforcement, genetic variants of DRD2 (rs1800497), and energy intake in individuals with obesity and non-obese humans and observed that energy intake was higher in individuals with high food reinforcement and even greater in those carriers of the TaqI A1 allele. Additionally, Rivera-Iñiguez et al. (2019) found a significant association between carriers of the Taq1a-A1 variant and overconsumption of unhealthy foods, especially meats, fried foods, sugars, and a decrease in legume consumption. Based on the analysis of nutrients, a higher consumption of carbohydrates was found. The variables we examined in our study were anthropometric variables, food addiction prevalence, and food reinforcing value, none of which showed such an association. It should be noted, however, that we found interesting associations between BMI and fat mass percentage in the overall sample. In the case of the reinforcing value, the association was observed only among heterozygous individuals. A variety of factors may affect the results of different studies, including differences in allelic frequencies, cultural differences among the populations studied, and different age groups. For example, Mexicans assign sentimental values to certain foods and processed foods are perceived as having a higher social status (Rivera-Iñiguez et al., 2019).

The creation of a bilocus genetic profile allowed us to study the aggregate effect of the two polymorphisms (rs1799732 and rs1800497), which individually have been associated with the dopaminergic system. Nikolova et al. (2011) previously found that MLGP for Dopamine signaling is relatively upregulated, predicting greater reward-related reactivity in the ventral striatum. Subsequently, Davis et al. (2013) found that the MLGP scores were significantly higher in the group of patients who were diagnosed with food addiction compared to control patients. Similarly, Yokum et al. (2015) and Obregón et al. (2022) found that participants with a higher number of alleles associated with high DA signaling capacity showed greater weight gain than those with a lower number of risk alleles. All these authors agree on how the MLGP was calculated, where each polymorphism was assigned a value of 0, 0.5, or 1, for the low, intermediate, and high dopamine signaling.

Finally, these scores at each locus for each participant are added to calculate a total individual profile score, allowing the inclusion of polymorphisms with non-significant independent effects. Using this approach, the bi-locus score was calculated, and we found that 12.7% and 1.4% of the sample presented high and very high dopaminergic signaling, respectively, and it was observed that those participants with more body fat, body weight, BMI, weight to height ratio (WH), and abdominal circumference (AC) presented trends toward higher dopaminergic signaling; however, these differences did not reach significance.

Our study observed no relationship between dopaminergic signaling and food reinforcement and food addiction. These data differ from a study conducted on 120 adults between 25 and 47 years of age in which the multilocus genetic profile score and food addiction were evaluated, incorporating hedonic impulse eating, binge eating, emotional eating, food cravings, and eating sweet snacks. To estimate the MLGP, they were based on six polymorphisms related to DA (Taq1A, -141C ins/del, Dat1, Val158Met, C957T, and rs12364283), finding a higher MLGP in those participants with food addiction, suggesting that the higher the MLGP produce lower dopaminergic signaling, leading to a greater response to palatable foods, causing food addiction (Davis et al., 2013).

There are many strengths and limitations to this study. As a result of the small sample size, some of our null findings regarding the relationship between rs1799732 and rs1800497 dopamine alleles and food addiction and eating behavior may have been due to the small number of adults with the diagnosis. This is very important because it is crucial to consider the ancestry of Latin American populations, since a low frequency of the risk allele may not permit association studies, because of differences between Amerindian, Caucasian, or African descent. According to previous research, the Chilean population has an average genetic contribution of 44.7% (Native American), 52.2% (European), and 3.01% (African). Furthermore, Bio Bio Region (origin of the sample) has previously reported 42.4% Native/Americans, 55.6% Europeans, and 1.9% Africans (Eyheramendy et al., 2015).

Also, this null association could be explained by the fact that individual polymorphic loci normally contribute only a small proportion of phenotypic variance and that their independent effects are typically not statistically significant. A multilocus genetic risk profile based on multiple DA SNPs may be more predictive of eating behavior and obesity than a bilocus genetic risk profile.

Despite these limitations, the current study represents the first to examine the relationship between bilocus score in dopamine genes, food reinforcement, and food addiction in Chilean populations. This study was conducted by a well-trained dietitian to conduct the surveys using face-to-face interviews.

## Conclusion

The results indicate that genetic variants rs1799732 (-141C Ins/Del) and rs1800497 (Taq1A) were associated with anthropometric measurements, especially in the heterozygous groups, but no association with food addiction was observed in Chilean university students.

In the group classified as “very high risk”, a trend toward overweight and obesity was observed, but this did not reach statistical significance. No significant differences were observed when bilocus scores were analyzed regarding eating behavior and food addiction. These results suggest that other genotypes, such as rs4680 and rs6277, which affect DA signaling capacity through a multilocus composite score, should be studied, either alone or in combination with those studied in the present study using an MLGP to more thoroughly evaluate the relationship between DA signaling, food addiction, and food reinforcement and energy intake.

## Data availability statement

The original contributions presented in the study are included in the article/Supplementary materials, further inquiries can be directed to the corresponding author.

## Ethics statement

The studies involving human participants were reviewed and approved by Comité Ético Científico Universidad San Sebastián. The patients/participants provided their written informed consent to participate in this study.

## Author contributions

NH and AO tabulated literature information. NH, KO, MV, GG, EG-G, and AO prepared the figures and improved the manuscript. NH, KO, and AO wrote the manuscript. All authors contributed to the article and approved the submitted version.

## Footnotes

^1^ http://www.uss.cl

## Abbreviations

DA: Dopamine
DRD2: Dopamine 2 receptor
BMI: Body Mass Index
FRVQ: Food Reinforcement Value Questionnaire
YFAS: Yale Food Addiction Scale
SNP: Single Nucleotide Polymorphisms
ANKK1: Ankyrin repeat domain containing 1 gene.
