# Supplementary material for: No association of the dopamine D2 receptor genetic bilocus score (rs1800497/rs1799732) on food addiction and food reinforcement in Chilean adults
Source: Front Behav Neurosci. 2023 Mar 31;17:1067384. doi: 10.3389/fnbeh.2023.1067384 (PMC10102336; doi:10.3389/fnbeh.2023.1067384)
Supplement: Supplementary file 1 [file Table_1.docx]

**Supplementary doc.**

**Table 1. Anthropometric measurements by carrier non-carrier status of rs1799732 and rs1800497 variants in females.**

|  |  |  |  |  | **Female** |  |  |  |  |
| --- | --- | --- | --- | --- | --- | --- | --- | --- | --- |
|  |  | **Normal** |  |  | **Over-weight** |  |  | **Obese** |  |
|  |  |  |  |  | **rs1799732** |  |  |  |  |
|  | GG  (n=60)  Mean± SD | G/Del  (n=17)  Mean± SD | *p*-value | GG  (n=11)  Mean± SD | G/Del  (n=4)  Mean± SD | *p*-value | GG  (n=44)  Mean± SD | G/Del  (n= 20)  Mean± SD | *p*-value |
| Weight (kg) | 57.8 ± 5.1 | 59.8 ± 5.3 | 0.2 | 66.8 ± 10.4 | 70.0 ± 6.2 | 0.43 | 87.2 ± 11.8 | 87.6 ± 10.1 | 0.59 |
| Height (mts) | 1.61 ± 0.05 | 1.62 ± 0.05 | 0.5 | 1.58 ± 0.08 | 1.58 ± 0.03 | 0.89 | 161 ± 0.05 | 158 ± 0.05 | 0.01* |
| BMI (kg/mt2) | 22.3 ± 1.49 | 22.9 ± 1.8 | 0.19 | 26.8 ± 3.2 | 28.3 ± 2.1 | 0.24 | 33.6 ± 4.3 | 35.2 ± 3.8 | 0.13 |
| Weight to height ratio | 0.47 ± 0.04 | 0.48 ± 0.04 | 0.3 | 0.55 ± 0.06 | 0.55 ± 0.06 | 0.89 | 0.65 ± 0.08 | 0.66 ± 0.09 | 0.7 |
| Abdominal circumference (cm.) | 74.8 ± 5.8 | 77.1 ± 6.9 | 0.19 | 87.1 ± 10.3 | 87.5 ± 9.95 | 0.94 | 105.1 ± 11.9 | 103.7 ± 13.8 | 0.62 |
| Body fat % | 26.3 ± 4.2 | 27.4 ± 4.1 | 0.27 | 33.5 ± 7.0 | 35.8 ± 3.3 | 0.43 | 44.1 ± 3.7 | 44.7 ± 4.20 | 0.39 |
| % Food Choice | 19.3 ± 21.5 | 17.1 ± 20.3 | 0.5 | 32.5 ± 30.3 | 8.3 ± 11.7 | 0.06 | 21.0 ± 25.1 | 21.2 ± 24.7 | 0.88 |
|  |  |  |  |  | **Female** |  |  |  |  |
|  |  | **Normal** |  |  | **Overweight** |  |  | **Obese** |  |
|  |  |  |  |  | **rs1800497** |  |  |  |  |
|  | A2/A2  (n=42)  Mean± SD | A1-Carrier  (n= 35)  Mean± SD | p-value | A2/A2  (n=8)  Mean± SD | A1-Carrier  (n=7)  Mean± SD | p-value | A2/A2  (n= 35)  Mean± SD | A1-Carrier  (n= 29)  Mean± SD | p-value |
| Weight (kg) | 57.3 ± 5.3 | 59.4 ± 5.0 | 0.07 | 69.3 ± 6.9 | 66.1 ± 12.1 | 0.56 | 87.2 ± 11.7 | 87.5 ± 10.8 | 0.7 |
| Height (mts.) | 1.61 ± 0.05 | 1.61 ± 0.05 | 0.93 | 1.56 ± 0.07 | 1.59 ± 0.06 | 0.35 | 1.6 ± 0.04 | 1.6 ± 0.06 | 0.4 |
| BMI(kg/mt2) | 22.0 ± 1.53 | 23.0 ± 1.46 | 0.004* | 28.3 ± 1.44 | 25.9 ± 3.81 | 0.08 | 34.0 ± 4.6 | 34.3 ± 3.8 | 0.85 |
| Weight to height ratio | 0.46 ± 0.03 | 0.48 ± 0.04 | 0.01* | 0.57 ± 0.03 | 0.53 ± 0.07 | 0.16 | 0.66 ± 0.09 | 0.65 ± 0.08 | 0.81 |
| Abdominal circumference | 73.6 ± 5.1 | 77.3 ± 6.7 | 0.02* | 89.6 ± 7.78 | 84.5 ± 11.9 | 0.64 | 105.4 ± 13.2 | 103.8 ± 11.6 | 0.68 |
| Body fat % | 25.7 ± 4.2 | 27.7 ± 3.8 | 0.02* | 36.3 ± 3.28 | 31.5 ± 8.0 | 0.16 | 44.3 ± 4.3 | 44.3 ± 3.3 | 0.89 |
| % Food Choice | 16.8 ± 18.6 | 21.1 ± 23.9 | 0.4 | 34.3 ± 34.9 | 16.6 ± 16.6 | 0.26 | 15.2 ± 20.6 | 28.1 ± 27.7 | 0.02* |
|  |  |  |  |  |  |  |  |  |  |
|  |  |  |  |  |  |  |  |  |  |

*Significant differences were analyzed with the nonparametric Mann-Whitney test by carrier status.

|  |  |  |  |  | **Male** |  |  |  |  |
| --- | --- | --- | --- | --- | --- | --- | --- | --- | --- |
|  |  | **Normal** |  |  | **Over-weight** |  |  | **Obese** |  |
|  |  |  |  |  | **rs1799732** |  |  |  |  |
|  | GG  (n=13 )  Mean± SD | G/Del  (n=7)  Mean± SD | *p*-value | GG  (n= 10 )  Mean± SD | G/Del  (n=0)  Mean± SD | *p*-value | GG  (n= 17)  Mean± SD | G/Del  (n= 9)  Mean± SD | *p*-value |
| Weight (kg) | 66.3 ± 6.9 | 71.7 ± 4.4 | 0.12 | 78.8 ± 7.5 | . | . | 99.3 ± 7.5 | 101.2 ± 12.4 | 0.74 |
| Height (mts) | 1.72 ± 0.06 | 1.76 ± 0.05 | 0.15 | 1.73 ± 0.07 | . | . | 1.74 ± 0.06 | 1.72 ± 0.05 | 0.51 |
| BMI (kg/mt2) | 22.4 ± 1.7 | 23.1 ± 0.9 | 0.32 | 26.3 ± 0.9 | . | . | 32.7 ± 2.1 | 34.0 ± 3.3 | 0.4 |
| Weight to height ratio | 0.46 ± 0.04 | 0.47 ± 0.02 | 0.4 | 0.51 ± 0.04 | . | . | 0.61 ± 0.04 | 0.59 ±0.06 | 0.5 |
| Abdominal circumference (cm.) | 78.8 ± 7.49 | 82.5 ± 4.2 | 0.15 | 88.1 ± 6.6 | . | . | 105.3 ± 6.4 | 102.0 ± 10.6 | 0.23 |
| Body fat % | 14.8 ± 3.6 | 15.0 ± 1.9 | 0.87 | 18.8 ± 3.8 | . | . | 29.7 ± 5.5 | 37.4 ± 6.1 | 0.008* |
| % Food Choice | 8.33 ± 8.33 | 8.33 ± 11.7 | 0.76 | 10.8 ± 18.8 | . | . | 21.0 ± 24.8 | 17.5 ± 16.9 | 0.9 |
|  |  |  |  |  | **Male** |  |  |  |  |
|  |  | **Normal** |  |  | **Overweight** |  |  | **Obese** |  |
|  |  |  |  |  | **rs1800497** |  |  |  |  |
|  | A2/A2  (n=12)  Mean± SD | A1-Carrier  (n=8)  Mean± SD | p-value | A2/A2  (n=7)  Mean± SD | A1-Carrier  (n=3)  Mean± SD | p-value | A2/A2  (n=15)  Mean± SD | A1-Carrier  (n= 10)  Mean± SD | p-value |
| Weight (kg) | 67. 7 ± 6.86 | 68.6 ± 6.3 | 0.78 | 78.0 ± 8.7 | 80.7 ± 4.6 | 0.81 | 98.4 ± 8.9 | 102.5 ± 9.8 | 0.18 |
| Height (mts.) | 1.73 ± 0.06 | 1.73 ± 0.04 | 0.78 | 1.72 ± 0.09 | 1.74 ± 0.03 | 0.9 | 1.74 ± 0.06 | 1.73 ± 0.05 | 0.55 |
| BMI(kg/mt2) | 22.5 ± 1.64 | 22.9 ± 1.3 | 0.75 | 26.1 ± 0.82 | 26.6 ± 1.2 | 0.56 | 32.6 ± 2.8 | 34.2 ± 1.9 | 0.06 |
| Weight to height ratio | 0.46 ± 0.04 | 0.46 ± 0.02 | 0.64 | 0.51 ± 0.05 | 0.51 ± 0.02 | 0.9 | 0.59 ± 0.06 | 0.61 ± 0.05 | 0.24 |
| Abdominal circumference | 80.3 ± 8.1 | 79.8 ± 4.0 | 0.78 | 88.0 ± 7.7 | 88.1 ± 4.3 | 0.9 | 103.1 ± 8.2 | 105.9 ± 8.2 | 0.34 |
| Body fat % | 14.6 ± 3.2 | 15.2 ± 3.1 | 1.0 | 18.6 ± 3.8 | 19.1 ± 4.7 | 0.7 | 31.8 ± 7.5 | 33.3 ± 5.9 | 0.73 |
| % Food Choice | 8.33 ± 8.7 | 8.33 ± 10.9 | 0.87 | 14.2 ± 21.9 | 2.7 ± 4.8 | 0.52 | 23.3 ± 25.6 | 16.6 ± 16.2 | 0.63 |
|  |  |  |  |  |  |  |  |  |  |
|  |  |  |  |  |  |  |  |  |  |

**Table 2. Anthropometric measurements by carrier non-carrier status of rs1799732 and rs1800497 variants in males.**

*Significant differences were analyzed with the nonparametric Mann-Whitney test by carrier status.

Table 3. Foods categories associated with food addiction. Odds ratio (OR) of food addiction (FA) for anthropometric variable

|  | Food addiction | |  |  |
| --- | --- | --- | --- | --- |
|  | Yes | NO | p- value |  |
| Ice-cream | 32.6% | 25.5% | 0.32 |  |
| Chocolate | 73.4% | 50.5% | 0.004 |  |
| Apple | 4.0% | 8.1% | 0.33 |  |
| Muffins | 6.12% | 15.1% | 0.10 |  |
| Broccoli | 0% | 4.6% | 0.12 |  |
| Cookies | 33.3% | 26.1% | 0.32 |  |
| Cakes | 38.7% | 29.6% | 0.22 |  |
| Candys | 18.3% | 8.1% | 0.03 |  |
| Bread | 63.2% | 39.5% | 0.003 |  |
| Rolls | 22.4% | 15.7% | 0.2 |  |
| Lettuce | 4.0% | 11% | 0.12 |  |
| Pasta | 46.9% | 33.1% | 0.07 |  |
| Strawberries | 10.2% | 12.2% | 0.7 |  |
| Rice | 36.7% | 21.5% | 0.03 |  |
| Crackers | 14.2% | 7.56% | 0.14 |  |
| Chips (empanada) | 40.8% | 19.9% | 0.002 |  |
| Pretzel (sopaipilla) | 28.5% | 11.0% | 0.002 |  |
| French Fries | 70.8% | 45.3% | 0.002 |  |
| Carrots | 4.9% | 0% | 0.15 |  |
| Steak | 8.1% | 5.2% | 0.15 |  |
| Bananas | 8.1% | 14.5% | 0.2 |  |
| Bacon | 51.0% | 24.4% | 0.001 |  |
| Hamburgers | 38.7% | 20.3% | 0.008 |  |
| Cheese burgers | 22.4% | 15.7% | 0.2 |  |
| Pizza | 44.9% | 34.3% | 0.1 |  |
| Soda | 40.8% | 26.6% | 0.04 |  |

P value < 0.05 * Chi-squared test.
